# Expression of CD68^+^ Cells in Synovial Tissue from Patients with PsA and its Association with Disease Activity Indices: A Clinical Pilot Study

**DOI:** 10.2174/0115733971314061241126044624

**Published:** 2024-12-03

**Authors:** Stanislava Popova-Belova, Mariela Geneva-Popova, Velichka Popova, Krasimir Kraev

**Affiliations:** 1 Department of Propedeutics of Internal Diseases, Medical Faculty, Medical University of Plovdiv, 4000, Plovdiv, Bulgaria

**Keywords:** CD68^+^ expression, psoriatic arthritis, gonarthrosis, synovium, high inflammatory activity, histological samples, tissue

## Abstract

**Introduction:**

Investigating CD68^+^ positive cells in the synovial tissue is crucial for understanding the pathogenesis of psoriatic arthritis (PsA) and developing targeted treatment strategies. The role of CD68^+^ positive cells in the synovial tissue of patients with PsA for joint destruction has not been fully studied.

**Objective:**

The objective of the study was to examine the presence of CD68^+^ cells in the synovial tissue of patients with PsA, particularly those with high inflammatory activity.

**Methods:**

Synovial tissue samples were collected during knee joint replacement surgeries from patients with PsA (16 patients) and gonarthrosis (25 patients). Immunohistochemical methods were employed to detect CD68^+^ cell expression in the tissue samples. The results were analyzed by histologists, and the staining intensity and percentage of positively stained cells were evaluated. The data were then divided into three groups for statistical analysis: negative, weakly positive, and strongly positive histological samples. Routine indices for disease activity, VAS, DAPSA, PASDAI, and mCPDAI were used to assess PsA activity in all patients and to assess correlations with CD68^+^ positive cells in the synovial tissue. Statistical analysis was performed using SPSS version 26.0 (SPSS Inc., Chicago, IL, USA).

**Results:**

The expression of CD68^+^ positive cells was significantly higher in patients with PsA compared to those with activated gonarthrosis (*p* < 0.001). The indices for disease activity, VAS, DAPSA, PASDAI, mCPDAI, and mCPDAI showed a significant positive relationship with the expression of CD68 + cells on synovial tissue in patients with PsA (*p* < 0.01)

**Conclusion:**

The findings of the study confirm the increased numbers of CD68^+^ cells in PsA *vs*. gonathrosis synovium. This suggests the need to explore therapeutic approaches aimed at suppressing or blocking CD68^+^ cells to potentially mitigate joint damage.

## 1 INTRODUCTION

Synovitis in psoriatic arthritis (PsA) is characterized by mucosal hyperplasia with an increased number of fibroblast- like synoviocytes and macrophages, hypervascularity, the presence of hyperemic forks, and subsynovial infiltrate of macrophages, lymphocytes neutrophils, and mast cells [1]. These cells in the synovial membrane lead to increased expression of tumor necrosis factor (TNF)-a, interferon (IFN) - gamma, interleukin (IL) -1, IL-2, IL-10, IL- 17, IL-18, and matrix metalproteinases [2]. Histological evaluation of psoriatic synovium showed that it is infiltrated with a significant amount of polymorphonuclear cells that produce chemo- kines, cytokines, enzymes, and metalloproteinases [3, 4]. The histological location of immune cells in synovial tissue in patients with PsA is heterogeneous and involves the presence of ectopic lymphoid structures. Proinflammatory cytokines (TNF-α, IL-6, IL-17) secreted by macrophages play a significant role in PsA [1, 5, 6].

CD68 is sialomucine, which is expressed by monocytes and macrophages, as well as subsets of CD34-positive hematopoietic stem cells, dendritic cells, neutrophils, basophils, and mast cells [1, 6, 7]. The presence of CD68^+^ is associated with the activation of these cells [1, 6, 7]. Alivernini *et al.* еxamined synovial tissue in patients to differentiate seronegative undifferentiated peripheral inflammatory arthritis [8]. The authors found that CD68^+^ cells are the most common mucosal cells in the studied patients with seronegative arthritis (*p* <0.001), and their study proved that the results of the histological examination of the synovium help in the diagnosis of undifferentiated peripheral arthritis [8].

Fiechter *et al.* (2021) investigated the impact of biological therapy on the treatment of PsA [9]. The authors used it as a marker of activity/suppressed activity of PsA CD68^+^sublinating macrophages, estimating their numerical reductions reaching statistical significance [9]. According to the authors, CD68^+^ cells play a significant role in inflammation and tissue damage associated with PsA [9].

According to Baeten *et al.*, infiltration of the synovial membrane with subsets of macrophages and polymorphonuclear cells reflects global disease activity in spondyloarthropathy, and similar results have been found in rheumatoid arthritis [10-12].

According to our previous publications, investigating correlations between disease activity indices and histological findings can provide crucial insights into the underlying mechanisms of PsA and potentially guide treatment strategies. Therefore, research looking for correlations between different clinical and histological indicators should be deepened [13].

The aim of the study was to examine the presence of positive CD68^+^ cells in the synovial tissue of patients with PsA.

## 2 MATERIALS AND METHODS

### 2.1 Characteristics of the Study and the Patients

This study was a retrospective case-control analysis that included 16 patients diagnosed with Psoriatic Arthritis (PsA) based on the CASPAR criteria and 25 patients diagnosed with gonarthrosis (GoA). The subjects underwent diagnosis and treatment in the Rheumatology Departments of the University General Hospital “St. George” and the University General Hospital “Kaspela” in Plovdiv, Bulgaria. The data collection occurred from July 2020 to August 2022. Only patients with Psoriatic Arthritis (PsA) who had asymmetrical joint involvement in several peripheral joints were included in the study. This group of patients was chosen since they made up the largest portion of individuals who sought medical care at the hospital throughout the recruitment period.

The inclusion criteria for the PsA patients were: (1) proven PsA arthritis with synovitis; (2) PsA non-treated with a biological agent (TNF-α-blocker, IL(interleukin)-17 blockers, Il-12/23 blockers); (3) absence of mental health comorbidities; and (4) signed informed consent form for participation in the study. The exclusion criteria for the PsA patients were: (1) refusal to give informed consent; (2) PsA treated with biologics; and (3) decompensated cardiovascular, pulmonary, or renal failure. The patients with gonarthrosis were with (ACR, 1991): (1) knee pain of at least 5 years, over 50 years of age, stiffness of less than 30 min, crepitations in the knee, deformity and enlargement of the joint, without warming; (2) radiographic evidence of osteophytosis of the knee joint; (3) erythrocyte sedimentation rate below <40 mm/h, negative rheumatoid factor; and (4) signed informed consent form for participation in the study. Excluded patients were with (1) the presence of crystalline arthropathy, (2) the presence of decompensated cardiovascular, pulmonary, renal, or hematological diseases, and (3) the presence of immunological phenomena. Through a biopsy, tissue material was taken and examined immunohistochemically. From the obtained material, 60 tissue samples were obtained from 16 joints of patients with PsA, and 100 tissue samples were obtained from 25 knee joints of patients with gonarthrosis.

The study was conducted in adherence to the World Medical Association Declaration of Helsinki (1964) and its revised version (Edinburgh, 2000). All work, including patient data analysis, blood collection and aspiration of synovial fluid, and the content of the informed consent form, was approved by the Committee for Scientific Ethics at the Medical University of Plovdiv, Protocol No 4/10.06.2021.

### 2.2 Procedure for Evaluation of Synovial Tissue

The preparations from synovial tissue were processed and prepared in the Immunological Laboratory of the Institute of Reproductive Biology at the Bulgarian Academy of Science, Sofia, and the results were analyzed by the Department of General and Pathological Anatomy, Medical Faculty, Medical University - Plovdiv. The histological results were analyzed by two independent histologists and, if necessary, when there was a difference in the result, by a third histologist, also known as an arbiter. At least 10 slides were made from each tissue sample and visualized by a specialist histologist. The following values are assumed for the intensity of the coloring: 0 - missing; 1 - weak; 2 - moderate; 3 - strong intensity. Strong intensity is reported as coloring of positive control and missing, as in negative control. For an adequate assessment of the staining reaction, all materials were compared with a negative control provided by the Department of General and Pathological Anatomy, Medical Faculty, Medical University - Plovdiv.

The percentage of positively stained cells was determined as follows: at 0-10% = 0; 10- 39% = 1; 40-69% = 2; 70-100% = 3. All cases in which the number of cells with positive staining was more than 10% were considered positive. For statistical analysis, according to the above scales, immunohistochemical results are divided into three groups (Table **1**) [14].

Paraffin sections, 4-5 μm thick, were mounted on adhesive slides and dried overnight at 37°C. Antigen detection was performed with citrate buffer (pH 6.0, cat. CPL500, ScyTek Laboratories, Inc., USA) for 20 minutes at 37°C. The obtained operative material was fixed in 10% neutral buffered formalin for 24 hours, after which samples with maximum dimensions of 1.0 x 1.0 cm were formed and fixed for another 24 hours. After dehydration was complete, the materials were placed in cedar oil for 2 to 4 days to extract the ethyl alcohol and clarify the material.

### 2.3 Documentation of Immunohistochemical Reactions and Processing of Digitized Images

After the preparations dried, the immunohistochemical reactions were examined and photographed with an Olympus BX 51 microscope equipped with an Olympus C5050Z MP camera (Olympus Optical Co. Ltd) in JPG format. The black and white dots on the graph were determined on each of the photos, and the contrast increased by 20%.

### 2.4 Quantitative Determination of the Intensity of the Reaction

To quantify the intensity of the immunohistochemical reaction, the gray density obtained from the conversion of the brown color reaction to black and white was measured using Adobe Photoshop v.7.01 (Adobe Systems Inc.) and Image Tool v.3.0 under Windows UTHSCSA, San Antonio, TX). Comparing the intensities of reactions between the individual sections was performed under the following requirements: reactions should occur at the same time, the test objects should be of the same slice thickness, and reactions between tissues of the same origin should be compared.

### 2.5 Disease Activity Indices

Based on the Visual analog pain score (VAS), the patients were categorized into three groups: patients with moderate pain (40-60 mm <), patients with severe pain (60-80 mm), and patients with very severe pain (>80-100 mm) [15].

According to the Disease Activity in PsA (DAPSA) score, disease activity was categorized into three groups: low activity ≤14; moderate activity>14 to ≤28, and high activity >28 [16].

According to Psoriatic Arthritis Disease Activity Score (PASDAI), disease activity categories were categorized into four groups: in remission <1.9; low activity >1.9 to 3.2; moderate activity >3.2 to 5.4; and high activity >5.4 [17].

On the basis of the modified Composite Psoriatic Disease Activity Index (mCPDAI), disease activity was categorized into three groups: low activity 1 to 3, moderate activity >3 to 9, and high activity > 9 [18-20].

### 2.6 Statistical Analysis

Statistical analysis was performed using SPSS version 26.0 (SPSS Inc., Chicago, IL, USA). Continuously measured variables were tested for normality through the Shapiro-Wilk’s test. The normally distributed data were described with means and standard deviations (SD), and comparisons were performed through an independent-samples t-test. Non-normally distributed variables were presented as medians and interquartile ranges (IQR) and analyzed through the Mann-Whitney U test. ROC analysis was used to construct an ROC curve to assess the overall diagnostic performance of the test and select an optimal cut-off value to determine the presence or absence of significance of the test. All tests were two-tailed, and the results were interpreted as significant at type error alpha = 0.05 (*p* < 0.05).

## 3 RESULTS

### 3.1 Demographic and Clinical Data about the PsA and GoA Patients

The PsA patients significantly comprise more men (n=11, 68.75%), with an average age of 57.17 ± 9.44, compared to the observed group of women (n=5, 31.25%) with an average age of 54.87 ± 4.56. Patients suffering from PsA have a significantly higher Body mass index (BMI) and a higher prevalence of diabetes, ischemic heart disease, hyperuricemia, dyslipidemia, and obesity in comparison to the patients with GoA (Table **2**). None of the patients had received intra-articular or systemic glucocorticosteroids for at least 36 months prior to the study. The crystals found in synovial fluid in patients with PsA were monosodium urate crystals (MSU).

### 3.2 CD68^+^ Cell Expression

Highly positive CD68^+^ cell expression (n = 60 samples, 100%) was observed in all synovial tissue samples from patients with PsA. In the synovial tissue of patients with gonarthrosis, there was a lack of positive expression of CD68^+^ cells (n = 100, 0%), and the difference between the two groups was significant (*p* <0.001) (Table **3**).

### 3.3 Immunohistochemical Analyses of Synovial Tissue in Patients with PsA

**Fig. (1):**
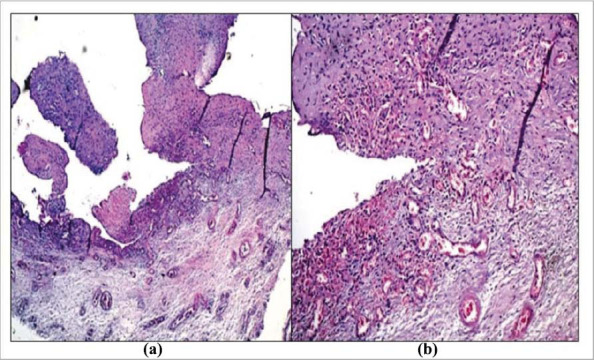
Immunohistochemical staining of CD68^+^, the sinovium of knee of patient with PsA, Panel (**a**) shows staining hematoxylin-eosin - moderately severe synovitis and granulation tissue at different stages of maturation. Panel (**b**) shows CD68-strong (+++) signalin monocyto- macrophage cells (x100 μm).

**Fig. (2):**
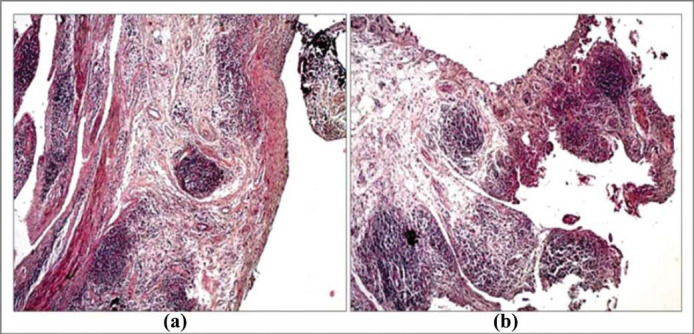
Immunohistochemical staining of CD68^+^, the sinovium of knee of patient with PsA, Panel (**a**) shows staining of hematoxylin-eosin-synovitis and granulation tissue in different stages of maturation. Panel (**b**) shows CD68 - strong (+++) signal in monocyto-macrophage cells (x 40 μm).

Immunohistochemical analyses of synovial tissue in patients with PsA differ significantly from those with gonarthrosis. The synovial tissue of patients with PsA was filled with many activated neutrophils, dendritic cells, synoviocytes, and macrophages. A sign of high activity of the listed cells was the presence of exposure to CD68^+^ (Figs. **1** and **2**).

Higher levels of CD68^+^ were observed in the group of patients with PsA (U_CD68_^+^ = 410.20, *p* <0.001). In patients with PsA, the median was 2, and the range was 0-3, while in patients with gonarthrosis, the median ranged from 0-1 in the range 0-3, with a significant difference in all activated cells observed (*p* <0.001) (Table **4**).

### 3.4 The Relation of Expression of CD68^+^ Cells on Synovial Tissue in Patients with PsA to Disease Activity Indexes

The VAS categories showed a significant positive association with the levels of expression of CD68^+^ cells on synovial tissue (r_s_ =0.856, *p* < 0.001) (Table **5**). According to the DAPSA, 7 (43.75%) of the PsA patients had moderate disease activity, and 56.25 (30.10%) were diagnosed with high disease activity. The DAPSA categories showed a significant, positive relationship with the expression of CD68^+^ cells on synovial tissue in patients with PsA (*p* < 0.001) (Table **4**). The PASDAI categories showed a significant positive relationship with the expression of CD68^+^ cells on synovial tissue in patients with PsA (r_s_ =0.669, *p* < 0.001). The mCPDAI categories showed a significant positive relationship with the expression of CD68^+^ cells on synovial tissue in patients with PsA (r_s_ =0.669, *p* < 0.001).

## 4 DISCUSSION

Interest in cell cytology in PsA-damaged tissues has been around for many years. The search for biomarkers that allow early identification of patients with severe, aggressive PsA and subsequent earlier treatment and better outcomes has recently increased interest in the study of inflamed synovial tissue in these patients.

We analyzed synovial tissue of patients with PsA, with a mean age of 54.36 ± 13.55 and BMI (34.49 ± 2.56), which are common for the disease, as noted by other authors [6, 15]. Patients have co-morbidities, as noted by other authors, which are not unusual for such patients [6]. During knee replacement surgery, synovial tissue was taken from patients for analysis because we wanted to investigate the presence of CD68^+^ in synovial tissue and its correlation with indices of disease activity. We found strongly positive CD68^+^ cell expression in all synovial tissue samples from PsA patients. We also found that in our samples of patients with gonarthrosis, there was a lack of expression of CD68^+^ cells in their synovial tissue, with the difference between the two groups being significant (*p* < 0.001).

Results similar to ours were also found by Mulherin *et al.* [11]. According to Mulherin *et al.*, CD68^+^ positive cells, which are macrophages, dendritic cells, and fibroblasts, accumulate in the synovium of arthritis patients and show destructive and remodeling potential, contributing significantly to joint inflammation and damage [11].

Haringman *et al.* and Mulherin *et al.* have shown that macrophage density in patients with RA and PsA correlates with disease activity [11, 12]. CD68^+^ macrophages have a destructive potential associated with their activation and production of enzymes, cytokines, and chemokines [11].

Alivernini *et al.* (2019) examined synovial CD68^+^ positive cells in patients with PsA and found that patients with higher levels of CD68^+^ had a poorer clinical response to therapy [2]. Alivernini *et al.* concluded that histological analysis of synovial tissue can help resolve the clinical overlap between PsA and RA and provide prognostic data for therapy success [2].

The CD68^+^ peptide is important for the phagocytic activity of tissue macrophages and is involved in intracellular lysosomal metabolism, as well as in interactions between isolated cells [10]. We found that the level of CD68^+^ was increased in the synovial tissue of patients with PsA compared with the synovial tissue of patients with activated gonarthrosis. As an expression of activation of macrophages, monocytes, fibroblasts, and dendritic cells, the CD68^+^ marker shows tissue expression more pronounced in patients with high clinical activity.

We investigated the relationship of CD68^+^ cell expression on synovial tissue in patients with PsA and disease activity by examining VAS, DAPSA, PASDAI, and mCPDAI and found a significant positive association of expression levels of CD68^+^ cells on synovial tissue with all indices of disease activity. Therefore, we assume that the presence of a high amount of CD68^+^ cells in synovial tissue is associated with high disease activity. We found no information on such correlations in the available literature.

We assume that it is likely that blocking CD68^+^ is associated with disease improvement. This would mean looking for a new therapeutic approach in patients with high disease activity in order to achieve clinical remission more quickly.

## CONCLUSION

Evaluation of the expression of CD68^+^ cells in the synovial tissue of patients with PsA proved their significantly stronger representation in the synovial tissue of inflammatory joint disease compared to degenerative joint disease. This is associated with more severe synovial destruction in PsA patients and, as such, may be used as a diagnostic biomarker. The relationship of expression of CD68^+^ cells in the synovial tissue of patients with PsA with indices of disease activity, including VAS for pain intensity, DAPSA, PASDAS, and mCPDAI, proves that higher disease activity is associated with stronger exposure of CD68^+^ cells and can be used as a prognostic biomarker.

## LIMITATIONS OF THE STUDY

The fact that the study was a single-centre retrospective study was its main limitation. Therefore, multicenter studies with larger samples are needed to validate the results of this study.

## AUTHORS’ CONTRIBUTIONS

The authors confirm their contribution to the paper as follows: study conception and design: MGP; data collection: SPB and KK; data analysis or interpretation: VP. All authors reviewed the results and approved the final version of the manuscript.

## Figures and Tables

**Table 1:** Results of immunohistological studies in patients with psoriatic arthritis.

Group	Characteristic	Description
1	Negative (-)	Includes all cells in which there is no staining or staining is up to 10% of the respective cells
2	Slightly positive (+)	Includes all cells in which the staining is with an evaluation of indicators from 1 to 2.
3	Strongly positive (+++)	Includes all cells in which the staining is with an evaluation of indicators above 2.

**Table 2:** Demographic and clinical data about the PsA and GoA patients.

**-**	**Groups**				
**Variables**	**PsA Patients**		**GoA Patients**		***p***
	***n***	**%**	**n**	**%**	
Sex Men* n* (%) Women* n* (%)	11 5	68,75 31.25	15 10	60 40	0.249^f^
Age (years), Mean ± SD Men Women	54.36 ± 13.55 57.17± 9.44 54.87 ± 4.56		71.46 ± 4.92		<0.001^t^
BMI, Mean ± SD	34.49 ± 2.56		28.14 ± 3.89		<0.001^t^
Comorbidity Hypertension Diabetes Ischemic h.d. Hyperuricemia Dyslipidemia Obesity	15 6 6 12 6 15	93.75 40.00 40.00 75.00 37.50 93.75	20 6 6 3 7 7	80.00 24.00 24.00 12.00 28.00 28.00	0.193^f^ <0.001^f^ 0.005^f^ <0.001^f^ <0.001^f^ <0.001^f^
Synovial fluid crystals	10	40	-	-	<0.001^f^

**Note:** ^t^—independent samples t-test; ^f^—Fisher’s exact test.

**Table 3:** Results from the ROC curve analysis regarding the ability of expression of CD68^+^ cells to discriminate PsA’s patients from GoA patients.

**Expression of** **CD68^+^ Cells**	**AUC 95% CI**	***P***	**Criterion** **Value**	**Sensitivity**	**Specificity**
PsA *vs*. GoA	1.00 (1.00 to 1.00)	<0.001	> 6	100%	100%

**Table 4:** CD68^+^ expression results positive on synovial tissue in patients with PsA and gonarthrosis (Mann-Whitney U test).

**Expression**	**Group**	**n**	**Medians (Range)**	**Mann-Whitney** **U**	**Significance** ***p***
CD68^+^	PsA	60	2 (0-3)	410.20	0.001
	Gonarthrosis	100	0 (0-2)		

**Table 5:** Results from the Spearman rank-order correlation analysis between disease activity indices and expression of CD68^+^ cells on synovial tissue in patients with PsA.

**Parameters** Expression of CD68^+^ Cells on Synovial Tissue in Patients with PsA	VAS	**DAPSA**	**PASDAI**	**mCPDAI**
● Correlation coef. rs ● 95% CI ● Significance (p)	0.856 (-0.07 to 0.21) 0.001	0.878 (0.19 to 0.31) 0.021	0.669 (0.14 to 0.23) 0.001	0.842 (0.05 to 0.22) 0.026

## Data Availability

All data generated or analyzed during this study are included in this published article.

## LIST OF ABBREVIATIONS

ACR: The American College of Rheumatology
AUC: Area Under the Curve
BMI: Body Mass Index
CASPAR criteria: Clasification Criteria for Psoriatic Arthritis
CV: Coefficient of Variation
DAPSA: The Disease Activity for Psoriatic Arthritis
ELISA: Enzyme-linked Immunosorbent Assay
GoA: Gonarthrosis
IL: Interleukin
IQR: Interquartile Ranges
mCPDAI: The Composite Psoriatic Disease Activity Index
PASDAS: The Psoriatic Composite Psoriatic Disease Activity Index
PsA: Psoriatic Arthritis
ROC curve: Receiver Operating Characteristic Curve
SD: Standard Deviations
SPSS: Statistical Package for the Social Science
TNF-α: Tumor Necrosis Factor-α
VAS: Visual Analogue Scale Measures Pain Intensity

## STANDARDS OF REPORTING

STROBE guidelines were followed.
